# Wastewater from healthcare centers in Burkina Faso is a source of ESBL, AmpC-β-lactamase and carbapenemase-producing *Escherichia coli* and *Klebsiella pneumoniae*

**DOI:** 10.1186/s12866-023-03108-0

**Published:** 2023-11-17

**Authors:** Zakaria Garba, Isidore O. J. Bonkoungou, Nadège O. Millogo, H. Magloire Natama, Pingdwendé A. P. Vokouma, Massa dit A. Bonko, Ibrahima Karama, Lagmêyesgo A. W. Tiendrebeogo, Kaisa Haukka, Halidou Tinto, Lassana Sangaré, Nicolas Barro

**Affiliations:** 1 Department of Biochemistry and Microbiology, Université Joseph KI-ZERBO, Ouagadougou, Burkina Faso; 2 Clinical Research Unit of Nanoro, Institut de Recherche en Sciences de La Santé, Nanoro, Burkina Faso; 3 Department of Microbiology and Human Microbiome Research Program, University of Helsinki, Helsinki, Finland; 4 Department of Health Sciences, Université Joseph KI-ZERBO, Ouagadougou, Burkina Faso

**Keywords:** ESBL, AmpC-β-lactamase, Carbapenemases, Multidrug-resistance, Hospital wastewater, *E. coli*, *Klebsiella pneumoniae*, Burkina Faso

## Abstract

**Background:**

Extended-spectrum β-lactamase (ESBL), plasmid-mediated AmpC-β-lactamase and carbapenemase-producing *Escherichia coli* and *Klebsiella pneumoniae* have spread into the environment worldwide posing a potential public health threat. However, the prevalence data for low- and middle-income countries are still scarce. The aim of this study was to evaluate the presence of ESBL, AmpC-β-lactamase and carbapenemase-producing and multidrug-resistant *E. coli* and *K*. *pneumoniae* in wastewaters from healthcare centers in Burkina Faso.

**Results:**

Eighty-four (84) wastewater samples were collected from five healthcare centers and plated on selective ESBL ChromAgar. *E. coli* and *Klebsiella pneumoniae* isolates were identified using API20E. ESBL-producing bacteria were detected in 97.6% of the samples and their average concentration per hospital ranged from 1.10 × 10^5^ to 5.23 × 10^6^ CFU/mL. Out of 170 putative ESBL-producing isolates (64% of them were *E. coli*) and 51 putative AmpC-β-lactamase-producing isolates, 95% and 45% were confirmed, respectively. Carbapenemase production was detected in 10 isolates, of which 6 were NDM producers, 3 were OXA-48 producers and 1 was NDM and OXA-48 producer. All isolates were multidrug resistant and, moreover, all of them were resistant to all tested β-lactams. Resistance to ESBL inhibitors was also common, up to 66% in *E. coli* and 62% in *K*. *pneumoniae.* Amikacin, fosfomycin and nitrofurantoin were the antibiotics to which the least resistance was detected.

**Conclusions:**

This study showed that wastewater from healthcare centers constitutes a reservoir of multidrug-resistant bacteria in Burkina Faso, including carbapenemase producers. Untreated healthcare wastewater entering the environment exposes people and animals to infections caused by these multi-resistant bacteria, which are difficult to treat, especially in the resource-poor settings.

## Introduction

The emergence and spread of antimicrobial resistance (AMR) represent a serious threat to human and animal health. In 2019, the number of deaths associated with AMR was estimated at 4.95 million including 1.27 million directly attributable to multi-drug resistant bacteria [1]. Therefore, if no action is taken against AMR, by 2050 this number could rise to 10 million per year [2, 3]. The economic cost of AMR has been estimated to vary from 1.1 to 3.8% of the global gross domestic product and the annual shortfall to reach $3.4 trillion by 2030 [4]. Several reasons, such as unreasonable use or overuse of antibiotics, have been speculated to favor the emergence and spread of resistance genes and multidrug resistant bacteria [5–10]. Furthermore, in low- and middle-income countries (LMICs), socio-economic and behavioral factors, such as poverty, use of poor quality antibiotics, absence of diagnostic tools, absence of antibiotic stewardship policies and uncontrolled use of antibiotics in animals, have been indicated [6]. The persistence of antibiotic residues, non-degraded antibiotics and disinfectants in wastewaters contribute to selection of resistant bacteria and their wide spread in environment [5–7, 11–15]. Multidrug resistant bacteria harboring extended-spectrum β-lactamase genes (*bla*_TEM_, *bla*_SHV_, *bla*_CTX-M_) and carbapenemase genes (*bla*_OXA-48_, *bla*_KPC_, *bla*_NDM_, *bla*_VIM_ and *bla*_IMP_) have been detected in hospital wastewaters from several countries [16–20]. Management of healthcare center wastewaters in LMICs is highly insufficient and sometimes the wastewaters are directly discharged into the environment, drainage, rivers, or lakes without any treatment [21–23]. Use of this water for various human activities exposes the population to new infections by multidrug resistant bacteria [24, 25].

In Burkina Faso, information on wastewater contamination is patchy, but recent studies have revealed the abundant presence of resistant bacteria in healthcare center effluents [26, 27]. The present study aimed to assess healthcare center wastewater contamination specifically by ESBL, AmpC-β-lactamase and carbapenemase -producing Gram negative bacilli and to perform phenotypic characterization of ESBL-producing *Escherichia coli* (ESBL-Ec) and *Klebsiella pneumoniae* (ESBL-Kp) in wastewaters from hospitals at different levels of the healthcare system in Burkina Faso.

## Results

### Bacterial concentration in healthcare center wastewaters

The average concentration of bacteria growing on ESBL-selective plates from wastewater of each healthcare center varied from 1.10 × 10^5^ to 5.23 × 10^6^ CFU/mL. The highest bacterial counts were obtained from wastewater drained from Yalgado Ouédraogo teaching hospital (tertiary level hospital), followed by Koudougou regional hospital center and El Fateh Suka clinic (both secondary level healthcare facilities) (Table 1).

**Table 1:** Average bacterial concentration from wastewaters of the five healthcare centers growing on ChromAgar™ ESBL plates

Healthcare centers	No. of samples (*n* = 84)	Average concentration (CFU/mL)
Yalgado Ouédraogo teaching hospital	28	5.23 × 10^6^
Koudougou regional hospital center	26	3.37 × 10^6^
El Fateh Suka clinic	14	3.00 × 10^6^
Source de Vie medical center	6	1.10 × 10^5^
Saint Camille medical center in Nanoro	10	1.85 × 10^5^

### Prevalence of ESBL

From the 84 healthcare center wastewater samples, ESBL *E. coli* or *K. pneumoniae* isolates were detected in 82 samples (97.62%). In total, 170 strains were isolated (109 *E. coli* and 61 *K. pneumoniae)*. ESBL test confirmed 160 (95%) bacterial isolates (102 *E. coli* and 58 *K. pneumoniae*) to be ESBL positive. ESBL confirmation test was negative for ten isolates but they were resistant to all the β-lactam and ESBL inhibitors tested.

### Prevalence of AmpC β-lactamase producers

Bacterial isolates with cefoxitin inhibition zone diameter less than 18 mm (37 ESBL-*Ec* and 14 ESBL-*Kp*) were tested to detect AmpC-β-lactamase production by the phenotypic method. In total, 23 out of 51 isolates tested (45%) were AmpC- β-lactamase producers (Table 2).

**Table 2:** AmpC-β-lactamase producers among isolates with cefoxitin inhibition zone diameter less than 18 mm from wastewaters of the five healthcare centers

**Healthcare centers**	***E. coli***		***K. pneumonia***		**AmpC-β-lactamase producers (%)** ^a^
	Tested (n)	AmpC positive (n)	Tested (n)	AmpC positive (n)	
Yalgado Ouédraogo teaching hospital	15	3	4	2	9.80
Koudougou regional hospital center	12	7	5	2	17.65
El Fateh Suka clinic	5	3	1	0	5.88
Source de Vie medical center	1	1	0	0	1.96
Saint Camille medical center in Nanoro	4	1	4	4	9.8
**TOTAL**	37	15	14	8	45.09

^a^ AmpC-β-lactamase-producing *E. coli* and *K. pneumonia* out of the 51 isolates tested

### Prevalence of carbapenemase producers

Twenty-one bacterial isolates resistant to meropenem (15 ESBL*-Ec* and 6 ESBL-*Kp*) were tested to detect carbapenemase production (OXA-48, KPC, NDM, VIM, and IMP). Ten isolates (47.62%) were carbapenemase producers: 6 were NDM producers, 3 were OXA-48 producers, and 1 was NDM and OXA-48 producer. Carbapenemase-producing bacteria were detected among wastewaters collected from the tertiary and the secondary level healthcare facilities (Table 3).

**Table 3:** Carbapenemase producers among the meropenem resistant isolates from wastewaters of the five healthcare centers

Healthcare centers	*E. coli*		*K. pneumoniae*	
	Tested (n)	Carbapenemase positive (n)	Tested (n)	Carbapenemase positive (n)
Yalgado Ouédraogo teaching Hospital	8	1 OXA-48 1 OXA-48 + NDM	4	2 OXA-48 1 NDM
Koudougou Regional hospital Center	4	1 NDM	1	1 NDM
El Fateh Suka clinic	3	2 OXA-48 1 NDM	0	0
**Source de Vie medical center**	0	0	0	0
Saint Camille medical center (Nanoro)	0	0	1	0

### Resistance to antibiotics

All the bacterial isolates from the ESBL selective plates (109 *E. coli* and 61 K*. pneumoniae* isolates) were tested against 31 antibiotics representing different antibiotic categories (Table 4). All the bacterial isolates were multidrug resistant. All the isolates (100%) were resistant to aminopenicillins (ampicillin, piperacillin) and cephalosporins except cefoxitin. In case of the ESBL-inhibiting combination antibiotics, 65.42% and 65.74% of *E. coli* and 61.67% and 45.76% of *K. pneumoniae* were resistant to amoxicillin + clavulanic acid and to piperacillin + tazobactam, respectively.

**Table 4:** Antibiotic resistance of ESBL-producing *E*. *coli* and *K. pneumoniae* strains

Antibiotic group	Antibiotics (concentration in µg)	Resistance to the antibiotic	
		***E. coli***	***K. pneumoniae***
		**n (%)**	**n (%)**
**Penicillin,** **penicillin and inhibitors**	Ampicillin (10)	97(100)	61(100)
	Piperacillin (100)	97(100)	60(100)
	Amoxicillin + acid clavulanic (30)	71(65.74)	37(61.67)
	Piperacillin + Tazobactam (110)	70(65.42)	27(45.76)
**Cephalosporin**	Cefazolin (30)	97(100)	60(100)
	Cefuroxime (30)	95(100)	56(100)
	Ceftriaxone (30)	95(100)	60(100)
	Ceftazidime (30)	95(100)	60(100)
	Cefepime (30)	95(100)	55(98.21)
	Cefoxitin (30)	43(40.57)	14(23.73)
**Monobactam**	Aztreonam (30)	92(94.85)	53(94.64)
**Carbapenem**	Meropenem (10)	17(15.74)	5(8.19)
	Imipenem (10)	22(20.75)	5(8.19)
	Ertapenem (10)	35(32.71)	11(18.33)
**Aminoglycosides**	Gentamycin (10)	46(44.66)	31(5082)
	Amikacin (30)	7(6.93)	8(13.11)
	Tobramycin (10)	74(71.15)	35(57.37)
	Kanamycin (30)	65(71.43)	35(77.77)
**Macrolides**	Azithromycin (15)	68(68.69)	21(35.59)
**Quinolones, fluoroquinolones**	Ciprofloxacin (5)	98(95.15)	56(91.80)
	Ofloxacin (5)	59(67.05)	11(24.44)
	Levofloxacin (5)	71(71.72)	32(53.33)
	Pefloxacin	58(100)	57(93.44)
	Nalidixic acid (30)	99(94.29)	40(88.89)
	Norfloxacin (30)	68(80.95)	30(50.0)
**Cyclins**	Tetracycline (30)	80(86.02)	36(78.26)
	Doxycycline (30)	70(67.31)	37(60.66)
**Sulfonamides**	Sulfamethoxazole (50)	73(93.59)	23(100)
	Sulfamethoxazole + trimethoprim (25)	94(89.52)	52(88.14)
**Nitrofurans**	Nitrofurantoin (300)	42(40)	24(40)
**Phosphonic acid**	Fosfomycin (200)	12(11.43)	36(61.02)

High resistance rates were detected against aminoglycoside, quinolone, and fluoroquinolone antibiotic categories. Indeed, in the aminoglycoside family, the detected resistance rates were up to 71.43% in *E. coli* and 77.77% in *K. pneumoniae* against kanamycin. Bacterial isolates were more susceptible to amikacin since only 6.93% of *E. coli* and 13.11% of *K. pneumoniae* were resistant (Table 4)*.* The resistance rates against quinolones and fluoroquinolones varied from 67.05% to 100% in *E. coli* and from 24.44% to 93.44% in *K. pneumoniae*.

In case of the carbapenems, 17 *E. coli* (15.74%) and 5 K*. pneumoniae* (8.19%) isolates were resistant to meropenem (Table 4).

Other families of antibiotics commonly used in hospitals in Burkina Faso include cyclins; 86.02% *E. coli* and 78.26% *K. pneumoniae* isolates were resistant to tetracycline. In case of the sulfonamides, 88.14% of *K. pneumoniae* isolates were resistant to sulfamethoxazole + trimethoprim and 100% to sulfamethoxazole (Table 4).

Azithromycin, an antibiotic widely used in Burkina Faso for Covid19 patient treatment [28, 29], was inactive against 68.69% of *E. coli* isolates and for 35.59% of *K. pneumoniae* isolates (Table 4).

## Discussion

Β-lactams are widely used in the treatment of patients in healthcare in Burkina Faso, but nowadays bacteria are often highly resistant to these antibiotics. Therefore, we isolated *E. coli* and *Klebsiella pneumoniae* strains from ESBL-selective ChromAgar plates inoculated with healthcare center wastewaters to determine their susceptibility to commonly used antibiotics. Over 97% of the 84 wastewater samples analyzed contained ESBL-producing *E. coli* and/or *K. pneumoniae*. The concentrations of ESBL-producing Gram-negative bacteria in the healthcare center wastewaters were high, but, our results are comparable to those published in previous studies in different parts of the world [16, 30–32]. For instance, concentrations up to 10^7^ CFU/mL of ESBL, CARB and OXA-producing Enterobacteriaceae were reported from hospital wastewaters in Slovenia and Austria [15]. Also in Burkina Faso’s neighboring countries, Ghana and Nigeria, ESBL-producers have been commonly isolated from hospital wastewater [33, 34] Among our samples, the wastewaters collected from the tertiary and secondary level healthcare centers were the most contaminated with ESBL producers, possibly because these hospitals receive more patients, generally referred from a district level healthcare. Also, antibiotics are used more in terms of both quantity and diversity in tertiary and secondary level hospitals.

In addition to being ESBL producers, many of the bacterial isolates characterized in this study were also AmpC-β-lactamase (23 positive out of 51 isolates tested) and carbapenemase (10 positive out of 21 isolates tested) producers. Two types of carbapenemases, (OXA-48 and NDM) were detected. Previously, using a metagenomics approach, presence of several carbapenemase genes (*bla*_VIM_, *bla*_IMP_, *bla*_NDM_ and *bla*_OXA-48_) was reported in the wastewaters of some of the same hospitals in Burkina Faso [27]. The resistance rate of our ESBL-producing *E. coli* and *K. pneunomia* isolates to carbapenems was 15.74% to meropenem, 20.75% to imipenem and 32.71% to ertapenem. Our results differ from those recently reported from Burkina Faso, where imipenem was the only carbapenem tested and no resistance to it was detected [26, 35]. Occurrence and eventual spread of the carbapenem resistant bacteria into the environment is of a particular concern since carbapenems are currently the antimicrobials of last resort in healthcare.

Wastewaters originating from healthcare centers present a public health concern in Burkina Faso and the other countries, where they are discharged directly into the environment or into municipality wastewater channels without any prior treatment [21, 23]. Furthermore, in LMICs, hospital wastewater may be used for irrigation of vegetable crops [5]. Indeed, ESBL-producing bacteria have been isolated on lettuce in Burkina Faso [35]. The common intestinal carriage of these bacteria may increase their prevalence in patients visiting healthcare centers, where the presence of these bacteria leads to complications of therapeutic treatment, prolonged patient hospitalizations and increased hospitalization costs, as well as higher mortality and morbidity [36].

High level of resistance to the commonly used antibiotics has been reported also by other research groups in West Africa [37, 38]. In Nigeria, full resistance to cefotaxime, cefpodoxime, sulfonamide and ertapenem was reported among ESBL-producing *E. coli* isolated from a healthcare facility wastewater [38]. Likewise, in Côte d’Ivoire, ESBL-producing *E. coli* and *K. pneumoniae* isolated from hospital wastewaters were reported to be fully resistant to amoxicilline + clavulanic acid, cefotaxime, ceftriaxone and ceftazidime. In addition, *E. coli* were fully resistant to ciprofloxacin, nalidixic acid and cefepime and *K. pneumoniae* were highly resistant to ciprofloxacin (62.5%), nalidixic acid and cefepime (87%) [37]. The high resistance level of bacteria in wastewaters from healthcare centers is a consequence of antimicrobial misuse in hospitals, the discharge at high concentrations of not metabolized antibiotics and antibiotic residues into hospital wastewater, and the fecal contamination by patients [8, 23, 39–42]. Furthermore, the high concentration of bacteria in these wastewaters offers an increased chance for horizontal transfer of resistance genes between bacteria [30–32, 37].

Amikacin, fosfomycin and nitrofurantoin were the antibiotics against which we recorded low resistance rates. Also in Mexico, a low resistance rate to amikacin among carbapenemase-producing *Klebsiella* spp. isolated from hospital wastewater was reported recently [43]. These antibiotics, mostly used for treatment of urinary tract infections, represent a major therapeutic option in case of infection with ESBL-producing bacteria.

## Conclusion

This study shows that wastewaters from healthcare facilities represent a reservoir of multidrug-resistant bacteria in Burkina Faso. Wastewaters collected from the healthcare centers representing tertiary and secondary level of the healthcare system were the most contaminated. The ESBL-producing *E. coli* and *K. pneumoniae* isolates were resistant to all commonly used antibiotics in Burkina Faso, such as β-lactams, β-lactams combined with ESBL-inhibitors (amoxicillin + clavulanic acid and piperacillin + tazobactam), quinolones, fluoroquinolones, aminoglycosides, sulfonamides, cyclins, and macrolides. Only amikacin and fosfomycin showed good activity against these bacteria. Some of the isolates also produced AmpC-β-lactamases and carbapenemases, limiting the treatment options even further. Untreated healthcare wastewaters entering the environment expose people and animals to the risk of infection by these multi-resistant bacteria. Therefore, it is important to include healthcare wastewater monitoring in the future national AMR monitoring program.

## Material and methods

### Study sites and sampling

A prospective study was carried out in 5 healthcare centers in Burkina Faso representing the different levels of the healthcare systemin Burkina Faso. The samples were collected from Yalgado Ouédraogo teaching hospital in Ouagadougou (university hospital, tertiary level care), Koudougou regional hospital center in Koudougou and El Fateh Suka clinic in Ouagadougou (secondary level care), Source de Vie medical center in Ouagadougou and Saint Camille medical center in Nanoro rural area (primary level).

Three healthcare centers had a sewer system, Yalgado Ouédraogo teaching hospital, Koudougou regional hospital center and Saint Camille medical center. Yalgado Ouédraogo teaching hospital sewers are connected to the city sewage system leading to the city’s wastewater treatment plant. Wastewater from the hospital is discharged into the general sewage without any prior treatment. Koudougou regional hospital center has a chemical treatment device. The treated wastewater is discharged into the municipality channel, which is connected to a backwater in the town. Source de Vie medical center and El Fateh Suka clinic do not have a sewer system and their wastewater is collected in septic tanks. The management of wastewater in these two healthcare centers and in Saint Camille medical center in Nanoro are not clearly documented. As a rule, there is no wastewater treatment plants in rural areas in Burkina Faso, instead, the wastewaters are directly discharged into the environment without any treatment.

We collected wastewater samples from several sites along the sewers from the healthcare centers with a sewer system and from septic tanks from the healthcare centers without a sewer system. Two rounds of sampling were done, 1) from october to december 2019 and 2) from october 2020 to march 2021. A total of 84 wastewater samples were collected (Table 1). In each case, one liter of wastewater was collected in a sterile glass bottle. The samples were immediately placed in a cooler containing ice blocks and transported within 12 h to the microbiology laboratory of the Clinical Research Unit of Nanoro (CRUN) for analysis.

### Bacterial count, isolation, and identification

Two dilutions were prepared for each sample (1/10 and 1/100) using sterile 0.9% physiological saline water. Following the WHO Tricycle instructions [44], 100 µL of each dilution was inoculated on ESBL-selective agar plates (ChromAgar™ ESBL, Paris, France), which were incubated at 35 ± 2 °C for 24 h. A positive control was carried out for all samples by inoculating a non-selective Cystine Lactose Electrolyte Deficient (CLED) agar plate with 100µL of the sample. After incubation, all visible bacterial colonies on the plates were counted, and the results were expressed into colony-forming units per milliliter of wastewater (CFU/mL). Only a plate of one dilution (1/10 or 1/100) from each sample was chosen for the bacterial count, depending on the abundance of bacteria on the plates.

The agar plates were also inspected for different morphotypes of bacteria, according to the manufacturer’s instructions (ChromAgar™ ESBL, Paris, France). Red or pink colonies were assumed to be *E. coli*, and blue, green, or blue-green the KESC group (*Klebsiella, Enterobacter, Serratia and Citrobacter*). Five colonies of the same morphotype of *E. coli* or the KESC group were picked for purification on eosin methylene blue agar (EMB). The purified isolates were identified using the API20E system (Biomérieux, Marcy-l’Etoile, France).

### Antimicrobial susceptibility testing

Antimicrobial susceptibility testing of 170 presumptive ESBL-producing bacterial isolates (109 *E. coli* and 61 K*. pneumoniae* isolates) was performed using the disk diffusion method on Muller Hinton (MH) agar. Thirty-one antibiotic discs (Condalab, Madrid, Spain) were tested (Table 4) and the results were interpreted according to the American Clinical and Laboratory Standards Institute (CLSI) 2021 guidelines [45].

### Extended spectrum β-lactamase (ESBL) confirmation

**Fig. 1:**
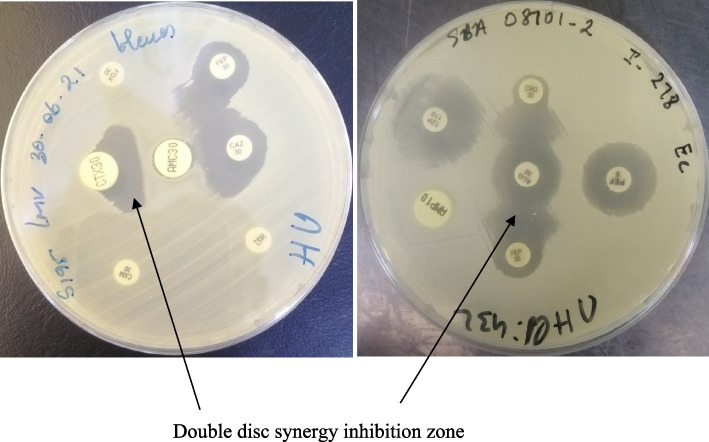
ESBL confirmation test for a *K. pneumoniae* strain showing a double disc synergy inhibition zone

ESBL confirmation was carried out on Mueller Hinton (MH) agar using the double disc synergy test (DDST) between a 3^rd^ generation cephalosporin (ceftriaxone or ceftazidime, C3G), a 4^th^ generation cephalosporin (cefepime, C4G) and amoxicillin + clavulanic acid (AMC), following the CLSI 2021 guidelines. The result was interpreted as positive when there was a visible synergy inhibition zone between C3G-AMC-C4G (Fig. 1).

### Phenotypic AmpC-β-lactamase testing

The 51 isolates (37 *E. coli* and 14 K*. pneumoniae*) with cefoxitin inhibition zone diameter less than 18 mm (≥ 18 mm) were tested for the AmpC-β-lactamase production. A bacterial suspension prepared with fresh colonies (McFarland 0.5) was inoculated onto entire surface of the MH agar supplemented with cloxacillin at 4 µg/l and a disk of cefoxitin was placed on the plate. The test was positive if the inhibition zone diameter around cefoxitin disc was ≥ 18 mm.

### Carbapenemases detection

The isolates that were resistant to meropenem were tested for carbapenemase-production using the immunochromatographic test O.K.N.V.I. RESIST-5 (CORIS BioConcept, Belgium) according to the manufacturer’s instructions. A total of 21 isolates were tested for the five main carbapenemases (OXA-48-like, KPC, NDM, VIM, IMP) within 15 min.

## Data Availability

All data generated or analyzed during this study are included in this published article.

## Abbreviations

AMC: Amoxicillin + clavulanic acid
AMR: Antimicrobial resistance
CAZ: Ceftazidime
CFU: Colony forming unit
CLED: Cystine Lactose Electrolyte Deficient
CLSI: Clinical and Laboratory Standards Institute
CTX: Cefotaxime
CRUN: Clinical research unit of Nanoro
C3G: Third generation cephalosporin
C4G: Fourth generation cephalosporine
DDST: Double disc synergy test
EMB: Eosin methylene blue
ESBL: Extended-spectrum β-lactamases
KESC: *Klebsiella**, Enterobacter, **Serratia* And *Citrobacter*
ITM: Institute of Tropical Medicine
MH: Muller Hinton
